# The training and support needs of 22 programme directors of community-based childhood obesity interventions based on the EPODE approach: an online survey across programmes in 18 countries

**DOI:** 10.1186/s12913-020-05709-1

**Published:** 2020-09-15

**Authors:** Jorien J. Slot-Heijs, Dorine C. M. Collard, Simone Pettigrew, Jan Vinck, Dennis Edell, Armando Barriguete, Tommy L. S. Visscher

**Affiliations:** 1 Mulier Institute, Herculesplein 269, NL-3584 AA Utrecht, The Netherlands; 2 The George Institute for Global Health, Level 5/ 1 King Street, Newtown, NSW 2042 Australia; 3 Hasselt University, Martelarenlaan 42, BE-3500 Hasselt, Belgium; 4 EPODE Canada, 20 Gothic Ave, Toronto, M6P 1T5 Canada; 5 National Institute of Medical Sciences and Nutrition, Vasco Quiroga, 14080 Tlalpan, Mexico; 6 Hanze University of Applied Sciences, Zernikeplein 7, 9747 AS Groningen, The Netherlands

**Keywords:** Community-based childhood obesity interventions, Need for training and support, Implementation, EPODE

## Abstract

**Background:**

Tackling childhood obesity is complex and requires a community-based approach implemented in multiple environments. It is known from literature that knowledgeable and skilled professionals are essential to implement such an approach successfully. The aim of the present study was to assess the need for training and support among a global network of programme directors implementing a Community-Based Childhood Obesity Intervention (CBCOI) based on the EPODE approach, in order to assist them in optimising the implementation process.

**Methods:**

An online survey was sent to 40 programme directors representing programmes implementing a CBCOI based on the EPODE approach. The survey consisted of statements on a 5-point Likert scale and multiple-choice questions about attitude towards and usefulness of training and support, and preferences for some predefined training types and training topics. In total, 22 programme directors responded to the survey (55% response rate). Data were analysed using descriptive statistics to describe the need for support in order to improve successful implementation.

**Results:**

Respondents strongly agreed that continually updating skills and learning how to make their programmes more effective and sustainable at the start and during the implementation was important. On-site training was preferred most at the commencement of a programme, while a 2-day training course was most valued during implementation. Monitoring, measuring and programme evaluation was identified as the most valuable training topic.

**Conclusions:**

The results indicate a continuing and significant need for support and training among programme directors implementing a CBCOI. The findings give directions regarding topics and types of training and support in order to improve the implementation process.

## Background

The prevalence of childhood obesity is increasing throughout the world [1, 2], and has been identified as a global challenge of epidemic proportions [3]. Children with obesity are at increased risk of impaired health during childhood, including higher risk of cardiovascular diseases, type 2 diabetes and negative psychosocial effects, such as poor self-esteem and depression [4]. Moreover, they are more likely to experience life-long obesity, which brings with it serious consequences for the individual, the healthcare system, and the economy [5–7]. It is therefore essential that governments and communities tackle childhood obesity.

Childhood obesity interventions must not only address factors at an individual level, but also address factors at an environmental level in order to be successful. Taking the environment into account is important, since the environment influences energy balance-related behaviours of individuals (physical activity and diet) [8]. Community-based interventions that target multiple environments, such as the home, the neighbourhood and the school, show promise in reducing childhood obesity [9]. More intense community-based interventions are related to lower childhood body mass index, in which intense interventions: (1) modified polices and systems (e.g. competitive pricing for food choices or establishing a walking path), (2) were continuous programmes rather than infrequent or a one-time event (duration) and (3) reached a greater proportion of the community population [10].

Successful implementation of health programmes, such as a Community-Based Childhood Obesity Intervention (CBCOI), is a complex process and has been shown to depend on a wide variety of implementation strategies, with different supposed mechanisms [11]. The degree of implementation of health programmes is associated with the nature of the programme (e.g. complexity and relevance for target group), the health professionals (e.g. knowledge and perceived social support), the organisation (e.g. staff capacity, performance management processes), and the socio-political context (legislation and regulations) [12]. Moreover, the assignment of clear roles and responsibilities, the provision of sufficient practical training and resources, and organisational support are crucial in the implementation of health programmes, such as CBCOI’s [13, 14]. Previous research suggests that feelings of disempowerment and inability among health professionals limit a successful implementation of programmes tackling childhood obesity [15].

In sum, to foster successful implementation there is a need to deliver qualified professional training and support to programme directors of CBCOI’s. The EPODE International Network (EIN) has delivered such support to CBCOI’s across the globe for the last decade. Some of these CBCOI’s are running for several years now and have developed into sustainable programmes in some countries [16].

### ‘Ensemble, Prévenons l’Obésité des Enfants’ (EPODE) International Network (EIN)

EIN is developed as a non-profit organisation with the aim to expand the scientific evidence base relating to childhood obesity prevention, to facilitate information sharing between individual programmes, and to foster links between relevant stakeholders across the public and private sectors [16, 17]. EIN also provides on-site training and support to the programme directors of individual programmes to assist them in the implementation of the EPODE approach. The EPODE approach is an evidence-based approach to childhood obesity prevention that has been shown to reduce childhood obesity by supporting communities in the creation of environments in which healthy behaviours become natural behaviours [15, 17–19]. The training and support that EIN offers to the programme directors consists of knowledge transfer of best practices via on-site training, formal (Obesity Forums) and informal meetings and via networking between programme directors. To date, the EPODE approach has been adopted in 28 countries worldwide (See Additional file 1 for an overview of programmes and countries). More information about the development and methodology of the EPODE approach is provided elsewhere [17, 20].

### Aim of the study

In a previous study conducted in 2014 barriers and facilitators to programme implementation were identified by means of questioning programme directors within EIN [16]. Programme directors appeared to face some critical implementation barriers for which they seek support, such as finding sustainable financial support, evaluation methods and developing communication strategies.

As empowering professionals is an essential element of CBCOI’s, the aim of the present study is to evaluate the need for training and support among programme directors implementing a CBCOI within the EIN.

## Methods

### Study frame and design

To assess the current needs for training and support, an online survey was developed for this study by EIN in collaboration with the Mulier Institute, an independent research institute in The Netherlands (see Additional file 2 for the survey). The Mulier Institute administered the survey among programme directors of CBCOI’s across the EIN. According to the national guidelines Medical Research Involving Human Subjects Act ethics clearance for the study was not necessary, as it did not involve medical scientific research and participants were not subjected to procedures or were required to follow rules of behaviour [21]. In the invitation for the study, the aim of the survey was clearly described and contact details were provided in case the respondents had any questions. Programme directors agreed with participating in the study by completing the survey.

### Measurements

Data was gathered by means of an online survey (see Additional file 2 for the survey). The first section of the survey asked respondents to provide information about background characteristics of their programmes, including geographical location. The second section of the survey was related to the attitude towards training and perceived usefulness of training options and topics using statements on a 5-point Likert scale. Respondents gave their preferences about additional support by a multiple-choice question and their willingness to pay for training services in a single-response question. Most questions were presented in multiple-choice format and were predefined by EIN.

### Sampling and procedures

A link to the online survey in English was sent to all 40 programme directors, representing 32 programmes associated with the EIN, in 28 countries (June 2017). A reminder was sent to those who did not complete the survey within 2 weeks. After 3.5 weeks, the data were extracted and analysed. A total of 26 programme directors started the survey, 22 of whom answered at least half of the questions and were included for analyses (55% response rate). These 22 respondents represented 19 programmes from 18 different countries.

### Data analyses

For three programmes two programme directors completed the survey. The sample size for responses relating to programme characteristics was 19 and the sample size for items relating to training and support requirements was 22. SPSS 20 was used for the statistical analyses. Data were analysed using descriptive statistics to describe the need for support in order to improve successful implementation. The analyses were performed by the Mulier Institute.

## Results

### Programme characteristics

Most of the programmes represented in the data were located in European countries (79%), with small numbers from Asia (Middle-East, 11%), North America (5%) and Australia (5%). Although European countries are strongly represented in the EIN (71%), programmes from European countries were somewhat overrepresented in the sample size.

Half of the CBCOI‘s were started between five and 10 years ago (53%). The other programmes started more than 10 years ago (21%) or less than 5 years ago (26%) (Table 1). Schools (47%) and municipalities (37%) were the most common primary settings for implementation of the programme. None of the programmes were primarily implemented in health care or after-school settings. Most programmes were controlled by a local authority (42%) (Table 1). All programmes involved professionals from at least two different policy domains in their programme, such as health care (84%), well-being (74%), and/or sports (74%). None of the programmes collaborated with professionals in the economy or safety sectors (Table 1).

**Table 1:** Characteristics of the CBCOI’s (*n* = 19)

	n	%
Year started		
> 10 years ago (2005–2008)	4	21
5–10 years ago (2009–2013)	10	53
< 5 years ago (2014–2018)	5	26
Main setting of implementation		
School	9	47
Municipality	7	37
Health care	0	0
After school	0	0
Other setting	3	16
Number of steering authorities		
1	12	63
2	4	21
3	2	11
4	1	5
Type of steering authorities (multiple-choice)		
National political authority	4	21
State or regional political authority	5	26
Local authority or branch of local government	8	42
School board	5	26
Other steering authority	8	42
Number of different policy domains involved in CBCOI		
2	1	5
3	6	32
4	5	26
5	3	16
6	2	11
7	1	5
8	1	5
Policy domains in which professionals implementing the CBCOI are involved (multiple-choice)		
Health care	16	84
Sports	14	74
Well-being	14	74
Parenting	13	68
Youth	10	53
Infrastructure/urbanisation	6	32
Mental health, youth addiction	3	16
Safety	0	0
Economy	0	0
Other policy domain	6	32

### Attitude towards training and improving skills

Almost all respondents endorsed the importance of updating skills and learning how to make their CBCOI more effective and sustainable (95% agree (of which 59% totally agree)) (Table 2). The majority of respondents agreed that management training should be a standard component of the programme budget (77% agree (of which 27% totally agree)). Most of the respondents feel that they need additional training, because the current guidance at an annual EPODE Obesity Forum is insufficient (73% agree (of which 5% totally agree)). Half of the respondents indicated that an external organisation should provide the training (50% agree (of which 9% totally agree)), most other respondents were neutral on this issue (41%).

**Table 2:** Programme directors attitudes towards training (*n* = 22)

	Totally disagree %	Disagree %	Neutral %	Agree %	Totally agree %
It is important for us continually update our skills and learn how to make our CBCOI more effective and sustainable	0	5	0	36	59
Management training, to achieve a solid foundation for all aspects of a CBCOI, should be a standard component of the programme budget	0	9	14	50	27
The guidance at the annual EPODE Obesity Forum is insufficient, we need additional training	0	5	23	68	5
A certification programme for our local and overall programme directors would give us and our financers confidence that we are on the right track	0	9	27	36	27
We organize our own training. However, we consider it a necessary expense to have someone else organize the training	9	0	41	41	9

### Training type options

Table 3 shows that when starting a CBCOI, 87% of the respondents considered on-site training for 3 to 5 days, followed by phone or email coaching, to be useful or very useful. An intensive 2-week training and an online training were also considered useful or very useful (77 and 64% respectively).

**Table 3:** Programme directors perceived usefulness of training options (n = 22)

	Not useful at all %	Not useful %	Neutral %	Useful %	Very useful %
**At the start of CBCOI**					
On-site training by one or two consultants– usually for a period of 3 to 5 days – followed by phone or email coaching	5	0	9	32	55
Participating in an intensive 2-week EPODE training programme at an associated institute, including interaction with the local programme and periodic follow-up over a 12-month period	9	0	14	18	59
Accessing an online training programme	0	0	36	9	55
**During the implementation of a CBCOI**					
Attending an intensive 2-day training workshop preceding an EPODE Obesity Forum	0	0	5	32	64
A course of training and workshops that would lead to a certification (diploma) as a CBCOI expert	0	0	14	14	73
Completing a programme audit followed by a custom training programme offered by 1 or 2 consultants at the site – usually for a period of 3 to 4 days on-site followed by telephone or email assistance	0	9	5	36	50
Accessing an online refresher training programme (MOOC)	0	0	23	32	45
Participating in an intensive weeklong EPODE refresher training programme at an associated institute, including: programme audit, interaction with the local programme communities and periodic follow ups over a 12-month period	9	0	18	9	64

Training during the implementation of a CBCOI was highly valued, since all proposed training options were regarded as useful by a majority of respondents (Table 3). A 2-day training session preceding the annual EPODE Obesity Forum (96% useful (of which 64% very useful)) and an expert certification course (87% useful (of which 73% very useful)) were valued most. Some training options were regarded as not useful or not useful at all by 9% of the respondents. Further analysis showed that these options were not consistently chosen by the same respondents.

All respondents regarded at least one of the suggested types of training, additional to the support EIN already provides, to be useful (Table 4). Training at EPODE Obesity Forums (73%) and a programme implementation and evaluation handbook (73%) were valued by the majority of respondents (Table 4), at a distance followed by a programme operational handbook for directors (64%). Some respondents mentioned other ideas (9%), including organised trips to best-practice community-based programmes within the network and meetings at the office of EIN (not in table).

**Table 4:** Additional support regarded as useful by programme directors (n = 22, multiple answers possible)

	%
More operational trainings at forums	73
A programme evaluation handbook / guide	73
A programme operational handbook for directors: process documentation (manual)	64
Regular online forums such as Webex for exchanging information with other programme directors	59
Online training packages	55
Programme self-audit with supporting documentation	50
Templates for reporting or budgeting	36
FAQ’s, e.g. a list of frequently asked questions	32
Other additional offerings	9
We do not need additional training	0

### Training topics

When asked to indicate desired training topics, all suggested potential training topics were considered useful or very useful by at least two-thirds of the respondents (Table 5). The five most preferred topics were: monitoring, measuring, and programme evaluation (96%), applying behaviour change theories for more effective intervention (95%), promoting and publishing programme results (95%), gaining parental involvement (91%), best practices for intervention design and delivery (91%) and designing built environments (91%).

**Table 5:** Programme directors attitude towards training topics (n = 22)

	Not useful at all %	Not useful %	Neutral %	Useful %	Very useful %
Monitoring, measuring and programme evaluation	0	0	5	23	73
Applying behaviour change theory for more effective interventions	0	0	5	36	59
Promoting and publishing programme results	0	0	5	59	36
Gaining parental involvement	0	5	5	32	59
Best practices in intervention design and delivery e.g. how to design a “drink more water” action	0	0	9	41	50
Designing environmental (built) environments	5	0	5	59	32
Assessing community needs and engaging community stakeholders	0	5	9	32	55
Fundraising techniques and proposal writing	0	0	14	36	50
Programme branding, obtaining local visibility and using social media to build online communities	0	9	5	41	45
Principles and practical application of social marketing	0	5	9	45	41
Skills for obtaining political commitment at all levels	0	5	14	36	45
Operational issues: annual plans, budgeting, hiring, motivating staff and volunteers	5	5	18	41	32
Managing public-private partnerships	0	5	27	27	41
Linking care (secondary prevention) and primary prevention	0	5	27	50	18

### Training cost

The willingness to pay for training services differed among respondents (not in table). One quarter of the respondents were willing to pay for services (23%), depending on the price. A total of 41% was unwilling to pay for training services, and 36% did not know whether they would be willing to pay. Responses related only to the concept of paying for training and not an absolute cost, as no estimate for a training budget was presented to the respondents.

## Discussion

The results of this study showed a continuing and significant need for support and training among programme directors implementing CBCOI’s based on the EPODE approach. There was a strong agreement among respondents regarding the importance of updating skills and learning how to make their programmes more effective and sustainable. This is regarded as important at the start of the implementation, as well as during the implementation process. Additional to the current support provided by EIN most programme directors valued more operational training and/or a programme evaluation handbook as useful. Training topics most often chosen as useful were ‘monitoring, measuring, and programme evaluation’, ‘applying behaviour change theory for more effective interventions’, and ‘promoting and publishing programme results’. Willingness to pay for training was limited, although most programme directors acknowledged that management training should be a standard component of the programme budget.

Reducing childhood obesity is complex and requires a long-term, multi-level approach [22]. When the multi-level approach requires environmental and policy changes, as per the EPODE approach, skills and knowledge about how to achieve those changes are needed at the start of the programme, but also later on a frequent basis as the programme matures. Therefore, it is not surprising programme directors express their needs at the start of the programme as well as during the implementation. Pettigrew and colleagues [16] concluded that programme directors need support in overcoming implementation barriers. Secure ongoing funding, standardized programme evaluation criteria and public-private partnership methods were identified as implementation barriers. These themes are in line with the topics programme managers state they need training and support for. Also, health professionals face challenges during the implementation of multi-level programmes, such as feelings of disempowerment in making environmental and policy changes, and an inability to work beyond traditional public health approaches. They need training in political and policy-making skills, and in establishing partnerships between the public and private sector [14]. This is also reflected in the present study, since almost all programme directors indicated a need for support with effective behaviour change techniques, obtaining political commitment and managing public-private partnerships.

Finances are a difficult issue. A total of 41% of the programme directors were unwilling to pay for training services. The unwillingness might be explained by the fact that training and support provided by EIN was free of charge until just before the start of the study. Possibly, programme directors did therefore not include budget for training. As a result of that programme directors did not have any training budget available.

The study showed common needs among programme directors. Some training topics, such as ‘monitoring, measuring and programme evaluation’, were regarded as useful by all programme directors. Apparently, EIN can provide the same type of training and support to all programme directors, despite the fact that their CBCOI’s have different characteristics and therefore require different implementation strategies. This is also despite the main variation in background regarding work experience and education of the programme directors. A possible explanation for this continuous need for support might be that the development of childhood obesity interventions rapidly changes over the years: from a focus on nutrition to physical activity to environmental factors and well-being. Programme directors might feel the need to keep updated about the developments in these fields too.

Limitations of the study were the response rate of 55% and the over-representation of European partner programmes. First, this might be due to the fact that the survey was provided in English, a possible barrier for some non-participants. Second, there might have been a decreased commitment from non-participants due to lack of resources in their programmes for training services. Therefore, programme directors in the non-response group may have felt it was not relevant for them to fill in the survey. Another limitation was the reliance on data from only programme directors from each programme. It is possible that other involved individuals (e.g. executive professionals) may have different needs for training and support. Finally, because we used an online survey with predefined response options and closed-end questions we were not able to probe respondents for further detail or seek clarification on the forms of support they need. A follow up qualitative study has been planned to build on this study and gather more contextual data on training and support needs.

The results of this study provides insight into the preferred type of training at the start and during the implementation and the preferred training topics of programme directors implementing a CBCOI. A needs assessment among programme directors is unique, since most needs assessments are focused on the target group of interventions [23, 24]. Moreover, many implementation evaluations are focused on identifying implementation barriers, but not on the types of training and support they need to overcome those barriers.

The results gives organisations such as EIN concrete directions for developing training and support types and topics for programme directors. Tailored training and support will foster the implementation process of an effective programme reducing the prevalence of childhood obesity. In the case of EIN this has led to the reformation of EIN partners into the international Youth Health Network (youthhealthcommunity.com). The next step is to allocate resources, as the results showed that not all programmes are willing to pay for training services. We feel confident that the results of the current paper will help among others prioritising the allocation of budgets.

## Conclusions

This study identifies a continuing and significant need for support and training among programme directors implementing a CBCOI. Programme directors strongly agreed that continually updating skills and learning how to make their programmes more effective and sustainable at the start as well as during the implementation was important. The most preferred type of training at the start was an on-site training, while a 2-day training course was most valued during implementation. Monitoring, measuring and programme evaluation was identified as the most valuable training topic. The findings give directions not only regarding topics, but also regarding types of training and support for programme directors in order to improve the implementation process of a CBCOI.

## Supplementary information

**Additional file 1.** Overview of programmes in the EPODE International Network.

**Additional file 2.** Online survey.

## Data Availability

The dataset used and/or analysed during the current study are available from the corresponding author on reasonable request.

## Abbreviations

CBCOI: Community-Based Childhood Obesity Intervention
EPODE: Ensemble, Prévenons l’Obésité des Enfants
EIN: EPODE International Network
