# Supplementary material for: The training and support needs of 22 programme directors of community-based childhood obesity interventions based on the EPODE approach: an online survey across programmes in 18 countries
Source: BMC Health Serv Res. 2020 Sep 15;20:870. doi: 10.1186/s12913-020-05709-1 (PMC7491184; doi:10.1186/s12913-020-05709-1)
Supplement: Supplementary file 1 — Additional file 1. Overview of programmes in the EPODE International Network. [file 12913_2020_5709_MOESM1_ESM.pdf]

Additional file I Overview of programmes in the EPODE International Network

NAME OF PROGRAMME	COUNTRY	PROGRAMME INFORMATION
Basord	Bulgaria	http://www.basord.com/
CamP Group	Germany	http://camp-group.org/en/
Cyprus Elementary School Intervention Program (CESIP)	Cyprus	Not available
ENERGIZE	New Zealand	https://www.sportwaikato.org.nz/programmes/team-energize.aspx
EPODE Flandre Lys	France	https://www.cc-flandrelys.fr/
Epode Umbria Region OBesity Intervention Study (EUROBIS)	Italy	https://www.eurobis.it/
GYERE program	Hungary	http://mdosz.hu/
Healthy Kids	Lebanon	https://youthhealthcommunity.com/meet-our-community/healthy-kids
Healthy Kids Bulgaria	Bulgaria	Not available
Healthy Kids Community Challenge	Canada	https://www.publichealthontario.ca/en/health-topics/health-promotion/child-youth-health/hkcc#:~:text=The%20Healthy%20Kids%20Community%20Challenge,health%20by%20promoting%20healthy%20behaviours.
Healthy Living Israel	Israel	Not available
Healthy Together Victoria	Australia	Not available
Healthy Traditions for Healthy Kids	Romania	Not available
Healthy Weight for Life	Malta	https://youthhealthcommunity.com/meet-our-community/healthy-weight-for-life
I'm Living Healthy Too! - SETS	Romania	http://sets.ro/ro/
ISCA	Denmark	http://isca-web.org/english/home
Jongeren Op Gezond Gewicht (JOGG)	The Netherlands	https://jongerenopgezondgewicht.nl/
Keep Fit	Poland	Not available
Montemorelos Adelante Con 5 Pasos	Mexico	Not available
MUN-SI	Portugal	https://mun-si.com/
Paideiatrofi	Greece	Not available
Petica-Play For Health	Croatia	https://youthhealthcommunity.com/meet-our-community/condimentum-purus-parturient-pellentesque
Project Spraoi	Ireland	https://projectspraoi.cit.ie/
RiseVT	United States of America	https://risevt.org/
Salud Madrid	Spain	Not available
Salzburg Together Against Obesity (SALTO)	Austria	https://www.salto-salzburg.at/
SCOPE	Canada	https://www.live5210.ca/
SPORTTUBE	Slovakia	Not available
SWEET	Northern Ireland	Not available
Tackling Childhood Obesity Together (TCOT)	United Kingdom	Not available
The Obesity Prevention and Lifestyle (OPAL) Program	Australia	https://www.sahealth.sa.gov.au/wps/wcm/connect/public+content/sa+health+internet/healthy+living/healthy+communities/local+community/opal/opal
Viasano	Belgium	http://www.viasano.be/nl/
