# Supplementary material for: The training and support needs of 22 programme directors of community-based childhood obesity interventions based on the EPODE approach: an online survey across programmes in 18 countries
Source: BMC Health Serv Res. 2020 Sep 15;20:870. doi: 10.1186/s12913-020-05709-1 (PMC7491184; doi:10.1186/s12913-020-05709-1)
Supplement: Supplementary file 2 — Additional file 2. Online survey. [file 12913_2020_5709_MOESM2_ESM.pdf]

Additional file II Online survey

1 Characteristics of the program

In this first section we ask you to describe the characteristics of the program in which you currently work. This will allow us to identify the support needs of specific kinds of programs. Note: if you are associated with more than one program, please choose the program with which you are most familiar.

Please provide contact information:

VR1 Program name

VR2 Your first and last name

VR3 Email address

VR4 Telephone number

VR5 Country

VR6 Commencement date of the program:

VR7 What is the *main* setting in which the program operates?

Label 1 Municipality (city, region, county)

Label 2 School(system)

Label 3 Health (hospital, primary care physicians, public health unit)

Label 4 After school setting (sport club, youth club, YMCA, …)

Label 5 Other, please specify: **VR7_ANW**

VR8 Approximately, how many children have been reached by your program to date?

Under which authority [1] is the program operating? (please select all that apply)

VR9A National political authority

VR9B State or regional political authority

VR9C The local authority or branch of local government

VR9D School board

VR9_AN Other, please specify: **VR9_ANW**

At what stage of the program is the target population directly involved? (please select all that apply)

VR10A Planning

VR10B Implementation

VR10C Evaluation of actions/interventions

VR10_AN Other, namely: **VR10_ANW**

VR10_GVB They are not involved in any stage

How is your target population involved? (please select all that apply)

VR11A Involved in the planning

VR11B Giving advice

VR11C Being consulted about plans

VR11D Providing resources

VR11E Program evaluation

VR11_AN Other, namely: **VR11_ANW**

Which stakeholder groups are actively involved in your community-based program? (please select all that apply)

VR12A Health Care Providers

VR12B Private sector partners - please specify: **VR13**

VR12C Local media

VR12D Sports clubs

VR12_AN Other, namely: **VR12_ANW**

People from which policy domains are actively collaborating in your community-based program? (please select all that apply)

VR14A Youth

VR14B Economy

VR14C Safety

VR14D Well-being

VR14E Health

VR14F Infrastructure/urbanization

VR14G Sports

VR14H Mental health, youth addiction

VR14I Parenting

VR14_AN Other, namely: **VR14_ANW**

How is your program funded? Please indicate the percentage of the total budget (please select all that apply)

VR15A Public government: ….%

VR15B Foundations …%

VR15C Research grants …%

VR15D Individual donations …%

VR15E Private (industry) …%

VR15F Other: namely: **VR15_ANW** ….%

If Private (industry) what percentage of private funding comes from:

VR16A Food industry: …%

VR16B Pharmaceutical: …%

VR16C Bank, insurance company: …%

VR16D Local business association …%

VR16E Other, namely: **VR16_ANW** ….%

VR16F I do not know

VR16G N/A

2. Use & satisfaction with current support.

At this moment the EPODE International Network (EIN) is already offering training and support. We would like to know how you feel about this support.

Did you use any <u>initial</u> training or support from EIN to operationalize the EPODE methodology? If so, please rate the usefulness of the support.

	Used?<br><br>**No=label 1**	Not useful at all **Label 2**	Not useful **Label 3**	Neutral **Label 4**	Useful **Label 5**	Very useful **Label 6**
Consultants visiting you on site **VR17A**	Yes/no					
Meetings with consultants at the EIN office. **VR17B**	Yes/no					
Visits to other EIN operating programs **VR17C**	Yes/no					
Workshops at an EPODE obesity forum **VR17D**	Yes/no					
Information from EIN publications and the EIN website **VR17E**	Yes/no					
Other: **VR17F** namely: **VR17_ANW**	Yes/no					

Did you use any <u>ongoing support</u> of EIN? If so, please rate the usefulness of the support.

	Used? **No=label 1**	Not useful at all **Label 2**	Not useful **Label 3**	Neutral **Label 4**	Useful **Label 5**	Very useful **Label 6**
Program Information via website **VR18A**	Yes/no					
Attendance at EPODE Obesity Forums (GOF, EOF, ECOF, …[2]) **VR218B**	Yes/no					
Presentations **VR18C**	Yes/no					
Formal workshops or webinars **VR18D**	Yes/no					
Networking with other program managers **VR18E**	Yes/no					
Phone(Skype) or email support from EIN coordinators **VR18F**	Yes/no					
Information from EIN publications and the EIN website **VR18G**	Yes/no					
Other: **VR18H** namely: **VR18_ANW**	Yes/no					

VR19 If relevant, cite an event or form of support that was particularly useful, and why.

VR20 If relevant, cite an event that was particularly disappointing, and why.

What additional offerings would you find useful? (please select all that apply)

VR21A A program operational handbook for managers: Process documentation (manual)

VR21B Program self-audit with supporting documentation

VR21C Online training packages

VR21D FAQ's e.g. a list of frequently asked questions

VR21E More operational training opportunities at Forums

VR21F Templates for reporting, budgeting etc.

VR21G A program evaluation handbook and guide.

VR21H Regular online forums such as Webex for exchanging information with other program managers

VR21_AN Other, namely: **VR21_ANW**

VR21_GVB All we need is available right now

3. Developing a new learning center

EPODE International Network (EIN) is considering establishing a formal training capability in collaboration with JOGG (the Netherlands). In this section we ask your opinion about some preliminary ideas for the support to be offered to both new and ongoing programs based on the EPODE Methodology:

Please rate the potential usefulness of these new training options for new programs:

	Not useful at all **Label 1**	Not useful **Label 2**	Neutral **Label 3**	Useful **Label 4**	Very useful **Label 5**
Participating in an intensive 2 week EPODE training program at JOGG (the Netherlands) or associated institute, including on site interaction with the JOGG program and periodic monthly follow ups over a 12 month period **VR22A**					
Accessing an online training program (MOOC – to be developed) **VR22B**					
Training by one or two consultants at your site – usually for a period of 3 – 5 days followed by phone or email coaching **VR22C**					

Please rate the potential usefulness of these training options for upgrading skills when you are already operating along the EPODE methodology:

	Not useful at all **Label 1**	Not useful **Label 2**	Neutral **Label 3**	Useful **Label 4**	Very useful **Label 5**
Participating in an intensive one week EPODE refresher training program at JOGG or associated institute, including: program audit, interaction with the JOGG program communities and periodic monthly follow ups over a 12 month period **VR23A**					
Accessing an online refresher training program (MOOC) **VR23B**					
Completing a program audit followed by a custom training program offered by one or two consultants at your site – usually for a period of 3 – 4 days on site followed by telephone or email assistance **VR23C**					
Attending an intensive two-day training workshop preceding an EPODE Obesity Forum **VR23D**					
A course of training and workshops that would lead to a certification (Diploma) as a community-based-program expert **VR23E**					

Please rate the potential usefulness of the following areas of training and support:

	Not at all useful **Label 1**	Not useful **Label 2**	Neutral **Label 3**	Useful **Label 4**	Very useful **Label 5**
Skills in obtaining Political commitment at all levels **VR24A**					
Program branding, obtaining local visibility and using social media to build communities online **VR24B**					
Applying behavior change theory for more effective interventions **VR24C**					
Assessing community needs and engaging community stakeholders **VR24D**					
Principles and practical application of social marketing **VR24E**					
Managing public-private partnerships **VR24F**					
Monitoring, measuring and program evaluation **VR24G**					
Linking care (secondary prevention) and primary prevention **VR24H**					
Designing Environmental (built) interventions **VR24I**					
Operational issues: annual plans, budgeting, hiring, motivating staff and volunteers **VR24J**					
Gaining Parental involvement **VR24K**					
Best Practices in intervention design and delivery e.g. how to design a “drink more water” action **VR24L**					
Promoting and publishing program results **VR24M**					
Fund raising techniques and proposal writing **VR24N**					
Other suggestions **VR24O**, namely: **VR24_ANW**					

4. Budget

VR25 Would your program be willing to pay for the services delivered by Epode at a cost-recovery basis?

Label 1 Yes, depending on the price and if it is affordable.

Label 2 No

Label 3 I do not know

VR26 Does your program have a training budget?

Label 1 Yes

Label 2 No

Label 3 I do not know

VR27 Approximately, how much money (euro) per year do you spend on average on training? .. euro

Label 999 I do not know

Please indicate how much you agree or disagree with the following statements

	Totally agree **Label 1**	Agree **Label 2**	Neutral **Label 3**	Disagree **Label 4**	Totally disagree **Label 5**
We organise our own training and do not consider it a necessary expense to have someone else do it. **VR28A**					
Training to achieve a sound foundation in all aspects of community-based program management should be included in every program budget. **VR28B**					
It is important for us to continually upgrade our skills and learn how to make our community-based program more effective and sustainable. **VR28C**					
We get all the guidance we need at the annual EPODE Forum. I can't see the need for additional training. **VR28D**					
A certification program for our local and overall program manager would give us and our funders confidence that we are on the right track. **VR28E**					
We appreciate the value of training but we're often too busy just running our program **VR28F**					

5. Useful existing instruments, facilities and documents

To improve the knowledge and skills in the field we are looking for good examples that are used in practice. In this section we ask you to inform us about such tools that could be shared with the network.

VR29 Do you use manuals, forms, reports, charts or other devices that are useful for daily practice and would you be willing to share these with other EIN programs?

Label 1 yes we use such devices, and I am willing to share

Label 2 yes, we use such devices, but they are proprietary and we cannot share them

Label 3 no, we do not use such devices

Label 4 I do not know

If yes, please provide contact information so we can contact you.

VR30A Same as 1a

if different from 1a:

VR31 Name / **VR32** Email address / **VR33** Telephone

6. Collaboration

VR34 We intend to collaborate closely with the EIN member programs in the development of an EPODE Academy. Would you be willing to be an EPODE Academy advisor and collaborate in the development of the EPODE Academy?

Label 1 Yes

Label 2 No

If yes, please provide contact information of a person in your organization who would be willing to actively contribute to this development:

VR35 Name

VR436 Email address

VR437 Telephone

7. Any remarks

VR39 Is there any other information or advice that you consider to be relevant and that is not covered by the above questions?

VR 40 Thank you for your time and contribution.

---

[1] By authority we mean the jurisdiction that is responsible for supporting the central coordination of your program.

[2] Global Obesity Forum (New York); European Obesity Fora: Amsterdam, Brussels, Lisbon, Bucharest; Epode Canadian Obesity Fora.
